# AlphaFold as a Prior: Guiding Protein Structure Prediction Using Experimental Data with ROCKET

**DOI:** 10.1063/4.0000860

**Published:** 2025-10-27

**Authors:** Alisia Fadini, Minhuan Li, Airlie J. McCoy, Thomas C. Terwilliger, Randy J. Read, Doeke Hekstra, Mohammed AlQuraishi

**Affiliations:** 1 University of Cambridge, Cambridge, Cambridgeshire, United Kingdom; 2 Harvard University, Cambridge, Massachusetts, USA; 3 New Mexico Consortium, Santa Fe, New Mexico, USA; 4 Columbia University, New York City, New York, USA

## Abstract

Advances in machine learning have transformed structural biology, enabling swift and accurate prediction of protein structure from sequence. However, challenges persist in capturing sidechain packing, condition-dependent conformational dynamics, and biomolecular interactions, primarily due to scarcity of high-quality training data. Emerging techniques, including cryo-electron tomography (cryo-ET) and high-throughput crystallography, promise vast new sources of structural data, but translating experimental observations into mechanistically interpretable atomic models remains a key bottleneck. Here, we address these challenges by improving the efficiency of structural analysis through combining experimental measurements with a landmark protein structure prediction method – AlphaFold2. We present an augmentation of AlphaFold2, ROCKET, that refines its predictions using cryo-EM, cryo-ET, and X-ray crystallography data, and demonstrate that this approach captures biologically important structural variation that AlphaFold2 does not. By performing structure optimization in the space of coevolutionary embeddings, rather than Cartesian coordinates, ROCKET automates difficult modeling tasks, such as flips of functional loops and domain rearrangements at low resolution. ROCKET does not require retraining of AlphaFold2 and is readily adaptable to other data modalities. This new type of structure refinement that optimizes latent representations in evolutionary space could unlock possibilities for high-throughput ligand screening, assemblies solved at low resolution, and conformational landscapes.

